# Polymer Inclusion Membranes with P507-TBP Carriers for Lithium Extraction from Brines

**DOI:** 10.3390/membranes12090839

**Published:** 2022-08-29

**Authors:** Xianjie Zeng, Li Xu, Tao Deng, Chengyi Zhang, Wei Xu, Wen Zhang

**Affiliations:** 1 State Key Laboratory of Chemical Engineering, Tianjin Key Laboratory of Membrane Science & Desalination Technology, School of Chemical Engineering and Technology, Tianjin University, Tianjin 300350, China; 2 Tianjin Mainland Hydrogen Equipment Co., Ltd., Tianjin 301609, China

**Keywords:** polymer inclusion membranes, P507-TBP carriers, lithium extraction, stability, high Mg/Li ratios

## Abstract

The separation of lithium and magnesium from salt-lake brines with high Mg^2+^/Li^+^ ratios is a main challenge for lithium extraction. In this work, novel polymer inclusion membranes (PIMs) were developed by incorporating 2-ethylhexyl phosphonic acid mono 2-ethylhexyl (P507) and tributyl phosphate (TBP) as the carriers into cellulose triacetate (CTA) polymers. The Li^+^ could be stripped from the P507-TBP extracting carriers using pure water eluents without adding concentrated hydrochloric acid, which can help decrease carriers’ leakage risk from membrane matrixes and keep the stability of PIMs. The morphology, composition, and wettability of P507-TBP-based PIMs were characterized systematically, and the carrier content in the PIM was also optimized. In the transport experiment with the feed of 0.1 mol/L LiCl and 4.0 mol/L MgCl_2_, the CTA/P507-TBP60% membrane exhibits a Li^+^ permeability of 4.76 × 10^−3^ mol·m^−2^·h^−1^ and a Li/Mg separation ratio of 10.2. After recycling seven times, the selectivity of the PIM is well-retained (>10), and the permeability of Li^+^ decreases slightly (less than 15%). With a decent selectivity and excellent stability, PIMs containing P507-TBP carriers show great potential for sustainable and efficient lithium recovery from brines with high Mg/Li ratios.

## 1. Introduction

Lithium, as a strategic metal element, has been widely used in energy storage materials [1,2]. Recently, the consumption of lithium and its compounds has rapidly increased with the widespread application of rechargeable lithium-ion batteries in electric vehicles and commercial electronics [3]. Lithium extraction from salt-lake brines is an effective way to meet the growing demand for industrial lithium resources owing to the low cost and large reserves [4,5]. However, due to their similar ionic properties, the efficient separation of Li^+^ and Mg^2+^ in the brines with a high Mg/Li ratio is the main obstacle to exploring lithium resources in salt lakes [6,7].

Many separation methods have been developed for lithium extraction from aqueous resources. Conventional methods, including solar evaporation, chemical precipitation, and solvent extraction, are constrained by complex processes and environmental pollution [8,9]. Recently, membrane separation technology, as a promising option for sustainable lithium extraction from brines with a high Mg/Li ratio, has attracted much attention due to its advantages of easy operation, high efficiency, and environmental protection [10,11,12,13]. Various membrane-based technologies for lithium and magnesium separation have been developed, such as electrodialysis [14], nanofiltration [15], diffusion dialysis [16], and liquid membrane extraction [17]. Among these technologies, lithium extraction by supported liquid membranes (SLMs) has also been extensively researched owing to its simple operation, high ion permeability, and selectivity [18]. However, the shorter service time of the membranes under operations due to the mass loss of extractants limits its further application [10,17]. Therefore, methods to develop novel liquid membrane technologies for efficient lithium extraction from brines are still needed. 

Polymer inclusion membranes (PIMs), as a promising type of homogeneous liquid membrane, have been considered as an attractive alternative to SLMs due to their better stability and less consumption of organic reagents [19]. PIMs are usually composed of a polymer matrix, an extraction carrier, and if necessary, a plasticizer [20]. The carrier encapsulated in the polymer matrix as the extraction agent is responsible for transporting the target species across the membrane [21]. PIMs have the advantages of high selectivity, eco-friendliness, easy preparation, and good mechanical properties [22,23,24]. Moreover, compared with solvent extraction, PIMs extraction is safe to handle and can achieve simultaneous extraction and stripping in one step [10]. These advantages have motivated numerous studies on the application of PIMs for extracting or removing metal species (e.g., Au(III) [25], Co(II) [26,27] Cu(II) [28], Zn(II) [29], Cr(VI) [23], V(V) [30]) from aqueous solutions, including Li ions. For example, Cai et al. [31] reported a CTA-based PIM with thenoyltrifluoroacetone and trioctylphosphine oxide as the carrier for the selective extraction of Li^+^ from solutions containing Na^+^ and K^+^. Cristhian et al. [32] embedded LIX-54-100 and Cyanex 923 into CTA and the obtained PIMs were applied to extract Li^+^ from seawater. Zhang et al. [33] employed the TBP/FeCl_3_ extractant to prepare PIMs, and the resulting membranes exhibited ultrahigh Li/Mg selectivity for lithium extraction from brines with a high Mg/Li mole ratio. However, the poor stability and durability due to the leakage of the carrier limits their application. Xu et al. [34] developed a stable ionic liquid-based PIM by incorporating TBP and 1-butyl-3-methylimidazolium bis(trifluoromethylsulfonyl)imide into CTA polymer for extracting Li^+^, but the selectivity of the PIMs is restricted. Hence, sustainable, stable, and highly selective PIMs still need to be developed for lithium extraction from salt-lake brines.

Here, a novel and efficient PIM containing tributyl phosphate (TBP) mixed with 2-ethylhexyl phosphonic acid mono 2-ethylhexyl (P507) as the extraction carrier in cellulose triacetate (CTA) polymer is developed and employed to extract Li^+^ from brines with a high Mg/Li mole ratio. Compared to other reported extraction systems (such as FeCl_3_-TBP), the Li^+^ in a P507-TBP extraction system could be easily stripped into the aqueous solution using only water instead of an acid solution [35], which is critical for improving the stability of the PIM membranes. The effects of the carrier content in the PIMs and the Mg/Li mole ratio in feed solution on the Li extraction performance of the PIM were studied in terms of permeability and selectivity. The PIM was also used for lithium extraction from synthetic brines, and the competitive effect of coexisting monovalent cations on the Li^+^ transport performance was also discussed. Moreover, the reusability was studied to evaluate the operational stability of the PIM. Finally, the transport kinetics of ions across the PIM was investigated by a long-term transport experiment to evaluate its potential for industrial application.

## 2. Experiments

### 2.1. Materials

Tributyl phosphate (TBP), CTA (degree of esterification: 61.3–61.9%), 2-ethylhexyl phosphonic acid mono 2-ethylhexyl (P507) and dichloromethane (DCM) were provided by Aladdin Chemical Co., (Shanghai, China). Lithium chloride (LiCl), sodium chloride (NaCl), potassium chloride (KCl), magnesium chloride hexahydrate (MgCl_2_⋅6H_2_O) and ferric chloride hexahydrate (FeCl_3_·6H_2_O) were purchased from Heowns Biochemical Technology Co., Ltd. (Tianjin, China). The other reagents were obtained from Kermel Chemical Reagent Co., Ltd. (Tianjin, China). All the reagents in this work were directly used for the experiment without further purification. The deionized water with a conductivity of less than 5 µS/cm was used as the medium to prepare the salt solutions. 

### 2.2. Preparation of PIMs

**Figure 1:**
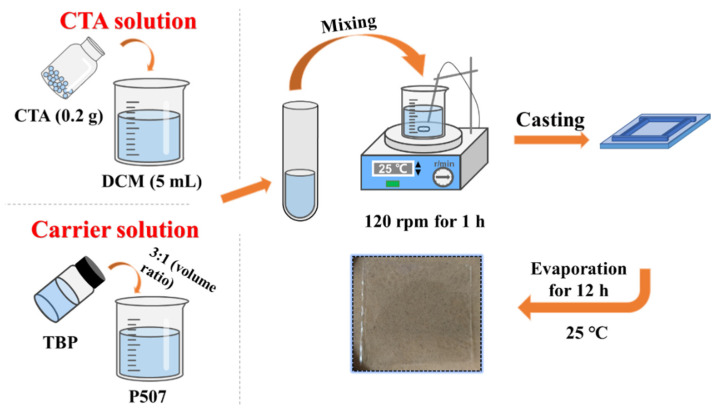
Schematic diagram of preparing CTA/P507-TBP membranes.

The PIMs using CTA as the polymer matrix and P507-TBP as extraction carrier were synthesized by the casting method according to the procedures in Figure 1. Specifically, 0.2 g of CTA was dissolved into 5 mL of DCM. The TBP and P507 were mixed uniformly with the volume ratio of 3:1 to prepare the extraction carrier [35]. Then the P507-TBP mixture was added to the CTA solution, and the homogeneous casting solution was obtained after stirring for 1 h. Finally, the casting solution was transferred onto a 5 cm × 5 cm flat glass and placed at room temperature for 12 h to evaporate the solvent. The resulting membrane was peeled and marked as CTA/P507-TBP*X*, where *X* refers to the mass fraction of P507-TBP in the membrane (*X* = 20 wt.%, 30 wt.%, 40 wt.%, 50 wt.%, 60 wt.% and 70 wt.%). The composition and thickness of PIMs are listed in Table 1. 

### 2.3. Characterization of PIMs

The surface and cross-sectional morphologies of CTA/P507-TBP membranes were observed by scanning electron microscopy (SEM, Hitachi S4800, Tokyo, Japan), and the element distribution of the membrane was investigated by energy dispersive X-ray spectroscopy (EDX, Tokyo, Japan). Fourier transform infrared spectroscopy (FTIR, Nicolet 6700, Massachusetts, America) with the scanning range of 800–2000 cm^−1^ was applied to study the structure of functional groups in PIMs. The water contact angle of the membrane surface was tested by an optical contact angle measuring analyzer (OCAH200). Five different sites of membrane sample were selected for the measurement of contact angles, and the average value was determined as the final result. 

### 2.4. Transport Experiments

**Figure 2:**
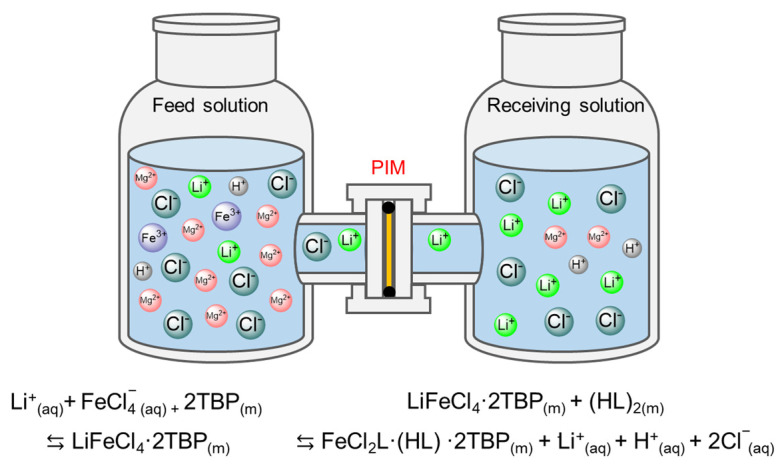
Schematic diagram of Li^+^ transport experiment: aq and m mean aqueous phase and membrane phase, respectively.

The transport experiments were performed in a two-compartment permeation cell to study the Li^+^ separation performance of the CTA/P507-TBP membrane. As shown in Figure 2, the feed side was filled with 150 mL of a mixed solution containing 0.1 mol/L of LiCl, 4.0 mol/L of MgCl_2_, 0.13 mol/L of FeCl_3_, and 0.03 mol/L of HCl. The mole ratio of Fe^3+^/Li^+^ in the feed solution was determined as 0.13 according to previous literature [35], and the presence of HCl was to inhibit the hydrolysis of Fe^3+^. The receiving side was filled with 150 mL of deionized water. The membranes were clamped between compartments with an effective area of 1.766 cm^2^. During the experiment, agitation was maintained on both sides to eliminate the effect of the concentration polarization. After terminating the experiment, the concentrations of Li^+^ and Mg^2+^ in the receiving solution were tested by inductively coupled plasma-optical emission spectrometry (ICP-OES, iCAP7000 series, Massachusetts, America).

The transport process of Li^+^ from the feed solution to the receiving solution across the CTA/P507-TBP membrane involves three typical steps: extraction, diffusion, and stripping. In the extraction process, TBP coordinates with Fe^3+^ and Li^+^ in the form of Li·2TBP·FeCl_4_, while P507 (HL) is not involved in the coordination. When the complex Li·2TBP·FeCl_4_ contacts with water at the interface between the receiving solution and PIM, the complex decomposes, and Li^+^ is successfully stripped into the receiving solution. On the other hand, P507 and TBP in PIM synergistically coordinate with Fe^3+^ to form the FeCl_2_L·(HL)·2TBP complex, and as a result, the Fe^3+^ was maintained in the PIM for the next extraction process.

The transport process of cations across the PIM conforms to the first-order reaction equation. The ions permeability coefficient (*P*, m/h) and initial flux (*J*_0_, mol·m^−2^·h^−1^) could be calculated by Equations (1) and (2) [36],

(1) $$\ln\left(\frac{C_{f,t}}{C_{f,0}}\right)=-\left(\frac{A}{V}\right)Pt$$

(2) $$J_{0}=P\cdot C_{f,0}$$

where $C_{f,0}$ (mol/L) is the ion concentration in the feed solution at the initial time, $C_{f,t}$ (mol/L) is the ion concentration in the feed solution at the sample time *t* (h), *A* (1.766 cm^2^) is the effective membrane area, and *V* (150 mL) represents the volume of the feed solution.

The separation factor (*SF*) of Li^+^ over other cations could be calculated by Equation (3),

(3) $$SF=\frac{C_{r,Li}/C_{r,M}}{C_{f,Li}/C_{f,M}}$$

where $C_{r,Li}$ is the receiving concentration of Li^+^, $C_{r,M}$ is the receiving concentration of M^n+^ (Mg^2+^, Na^+^ and K^+^), $C_{f,Li}$ is the feed concentration of Li^+^, and $C_{f,M}$ is the feed concentration of M^n+^.

## 3. Results and Discussion

### 3.1. Characterization of the PIMs

#### 3.1.1. Morphology Study

**Figure 3:**
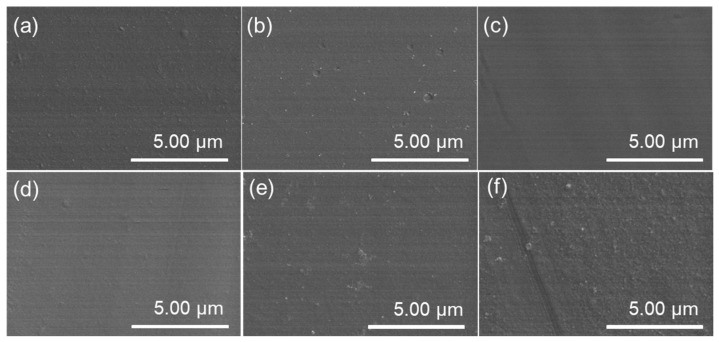
Surface morphologies of the PIMs with different content of carrier: (**a**) 20%; (**b**) 30%; (**c**) 40%; (**d**) 50%; (**e**) 60%; and (**f**) 70%.

**Figure 4:**
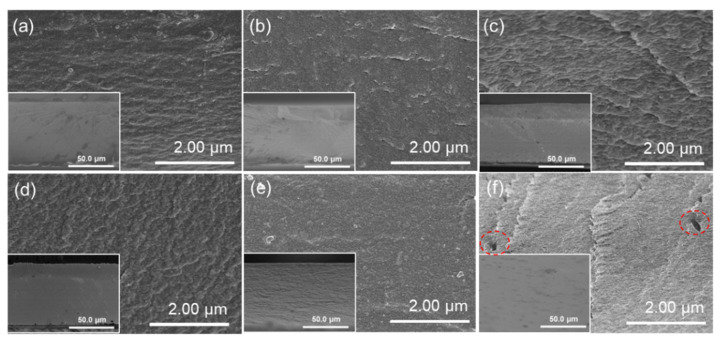
Cross-sectional morphologies of the PIMs with different content of carrier: (**a**) 20%; (**b**) 30%; (**c**) 40%; (**d**) 50%; (**e**) 60%; and (**f**) 70%.

The surface and cross-sectional morphologies of the developed PIMs were investigated by SEM images, as shown in Figure 3 and Figure 4. The surface and cross section of the PIMs with the carrier content ranging from 20 wt.% to 60 wt.% are compact, homogeneous, and without obvious holes or cracks, which indicates that the CTA polymer has excellent compatibility with the P507-TBP carrier. However, as the carrier content was further increased to 70 wt.%, there were many bubble-like protrusions on the membrane surface, and visible voids could also be observed on the membrane cross section. The presence of voids in the membrane will facilitate ion transport in these defects without selectivity. 

#### 3.1.2. FTIR and EDX Analysis

**Figure 5:**
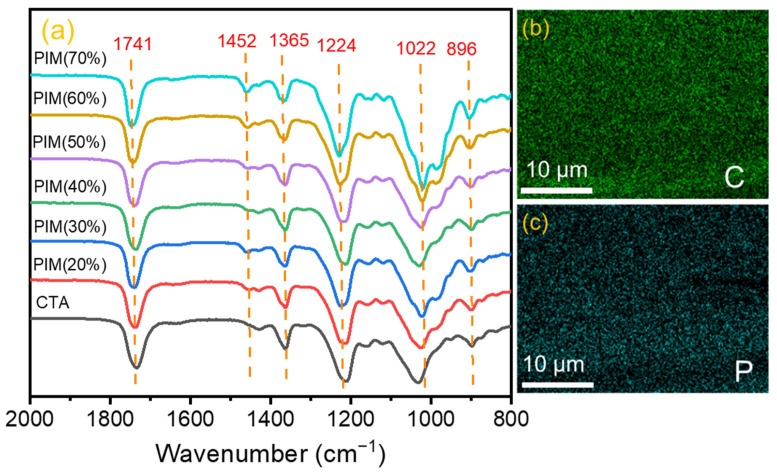
(**a**) FTIR spectra of CTA and the obtained PIMs; (**b**,**c**) element mapping of CTA/P507-TBP 60% membrane.

FTIR spectra of different PIMs are shown in Figure 5a, the absorption bands at around 1741 cm^−1^ and 1365 cm^−1^ are assigned to the stretching vibration of C=O and C-O bonds existing in the CTA polymer [37]. The bands located at 1224 cm^−1^ and 1022 cm^−1^ are attributed to the stretching vibration of P=O and P-O-C bonds existing in TBP [38]. The bands at around 896 cm^−1^ and 1452 cm^−1^ correspond to the stretching vibration of C-H and P=O bonds existing in P507, respectively [39]. All of the above characteristic bands are present in the FTIR spectra of CTA/P507-TBP membranes, indicating that the carrier (P507-TBP) was incorporated into the CTA matrix without chemical change. 

Furthermore, EDX analysis of the CTA/P507-TBP60% membrane was carried out to study the carrier distribution in the PIMs. As shown in Figure 5c, element mapping demonstrates that P is evenly distributed in the cross section of the membrane, while the P element exists only in P507-TBP, confirming the uniform distribution of the carrier and successful preparation of PIMs.

#### 3.1.3. Surface Hydrophilicity Analysis

**Figure 6:**
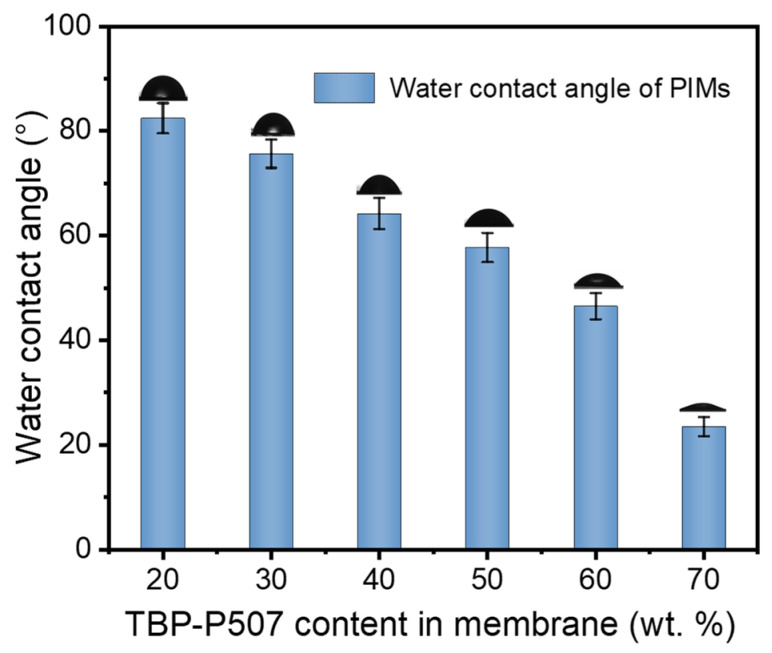
Water contact angles of the PIMs with different content of P507-TBP; *n* = 5.

Surface hydrophilicity considerably impacts the ion transport properties and stability of PIMs. Here, water contact angles of the PIMs with different carrier contents were measured to evaluate the surface hydrophobic/hydrophilic character. As shown in Figure 6, all of the PIMs investigated in this study exhibit a hydrophilic character since the contact angle values are less than 90° [40], and the hydrophilicity of the PIM is strengthened with the increased content of P507-TBP. The water contact angle value of the PIM decreases from 82.4° to 23.5° when increasing the carrier content from 20 wt.% to 70 wt.%. The more hydrophilic surface is conductive to achieve higher ion transport flux because the interaction between the target ions in solution and the carrier in the hydrophilic membrane is easier, thereby facilitating the process of ion transport across the PIMs. However, the extreme hydrophilicity may promote the leakage of the carrier, resulting in poor stability of the PIMs [21].

### 3.2. Separation Performance of the PIMs

#### 3.2.1. Optimization of the Carrier Content 

**Figure 7:**
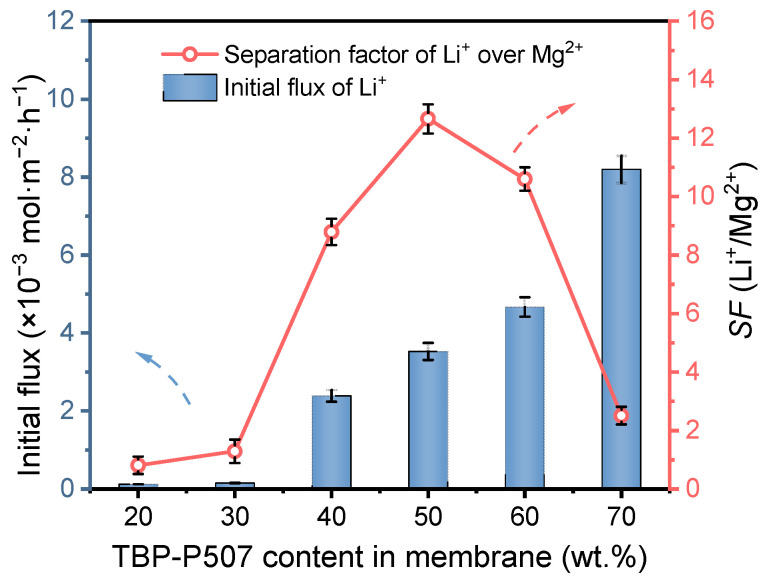
Li^+^ separation performance of the PIMs with different contents of carrier (P507-TBP). Feed solution: 0.1 mol/L LiCl; 4.0 mol/L MgCl_2_; 0.13 mol/L FeCl_3_; test time: 24 h; *n* = 5.

The extraction carrier is the most critical component of the PIM because it can determine the separation performance of the membrane. In order to optimize the carrier content in PIM, transport experiments with PIMs containing different amounts of P507-TBP were performed for 24 h. The initial flux of Li^+^ and the *SF*(Li^+^/Mg^2+^) were calculated, and the obtained results are shown in Figure 7. It can be observed that the PIMs with carrier content less than 30 wt.% exhibit little ability for Li^+^ selective separation from the aqueous solution. When increasing the carrier content from 40 wt.% to 70 wt.%, the initial flux of Li^+^ for the PIM shows an increasing trend, and the value for the CTA/P507-TBP 70% membrane reaches about 8.2 × 10^−3^ mol·m^−2^·h^−1^. The *SF*(Li^+^/Mg^2+^) of the PIM increases firstly and then decreases, and the maximum selectivity is obtained when the carrier content is 50 wt.% with the value of 12.67. Then the *SF*(Li^+^/Mg^2+^) of the PIM decreases sharply as the carrier content further increases to 70 wt.%. This can be explained by the voids in the membrane (SEM images in Section 3.1.1). Therefore, considering both permeability and selectivity, the 60 wt.% can be used as the optimal carrier content. The Li^+^ initial flux and the *SF*(Li^+^/Mg^2+^) of CTA/P507-TBP60% membrane is 4.67 × 10^−3^ mol·m^−2^·h^−1^ and 10.62, respectively. 

#### 3.2.2. Effect of Mg/Li Ratio and Coexisting Cations

**Figure 8:**
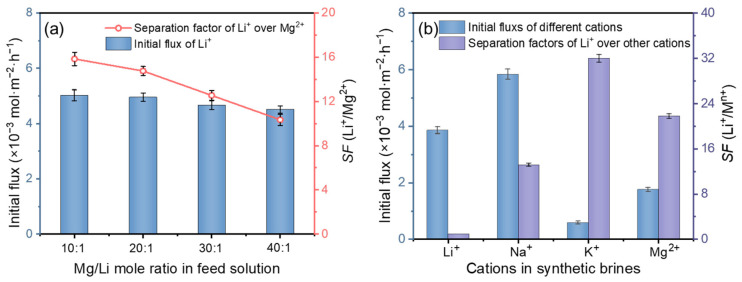
(**a**) Effect of Mg/Li mole ratio (feed solution: 0.1 mol/L LiCl, 1.0/2.0/3.0/4.0 mol/L MgCl_2_, 0.13 mol/L FeCl_3_); (**b**) Li^+^ separation performance from synthetic brines (feed solution: 0.1 mol/L LiCl, 2.0 mol/L NaCl, 0.5 mol/L KCl, 1.0 mol/L MgCl_2_, 0.13 mol/L FeCl_3_). Membrane: CTA/P507-TBP60%; test time: 24 h; *n* = 5.

There are obvious differences in the Mg/Li ratio of real salt-lake brines, and the high Mg/Li ratio has an unfavorable influence on the effective separation of Li^+^ and Mg^2+^ [41,42]. Here, transport experiments were carried out using feed solution with different Mg^2+^ concentrations, and the Li^+^ flux and *SF*(Li^+^/Mg^2+^) were evaluated to investigate the effect of the Mg/Li mole ratio on the separation performance of PIM. As shown in Figure 8a, with the increase of the Mg/Li mole ratio from 10 to 40, the selectivity of the CTA/P507-TBP membrane showed a declining trend, and the *SF*(Li^+^/Mg^2+^) decreased from 15.8 to 10.3. Simultaneously, the initial flux of Li^+^ decreased slightly from 5.02 × 10^−3^ mol·m^−2^·h^−1^ to 4.51 × 10^−3^ mol·m^−2^·h^−1^. These results confirm that the PIM developed here is suitable for effectively extracting lithium from high Mg-containing brines.

In addition, there are other coexisting cations (such as Na^+^ and K^+^) in natural brines, and the concentration of these cations may be higher than that of Li^+^, which can generate the competitive permeation with Li^+^ and thus affect the lithium extraction performance of the PIMs [43]. Herein, the CTA/P507-TBP membrane was also used to extract Li^+^ from synthetic brines containing Na^+^ and K^+^ as competitive cations, and the experimental results are shown in Figure 8b. The Li^+^ selectivity of the PIM was enhanced at the slight cost of the permeate flux when the feed brines contain Na^+^ and K^+^. With Na^+^ and K^+^ in the feed solutions, the *SF*(Li^+^/Mg^2+^) reaches as high as 21.8, with the Li^+^ initial flux of 3.86 × 10^−3^ mol·m^−2^·h^−1^. This is because the permeability coefficients of different cations across the PIM follow the order of Li^+^ > Na^+^ > Mg^2+^ > K^+^, and the competitive effect of Na^+^ on Mg^2+^ inhibits the migration of Mg^2+^ to a certain extent, thus promoting the effective separation of Li^+^ and Mg^2+^. On the other hand, the limited content of the extraction carrier is responsible for the slight decline of Li^+^ flux. Therefore, the PIM reported here exhibits excellent selectivity (Li/Na = 13.2, Li/K = 32, Li/Mg = 21.8) for extracting lithium from real salt-lake brines and has potential for industrial applications. 

#### 3.2.3. Stability and Reusability of the PIMs

**Figure 9:**
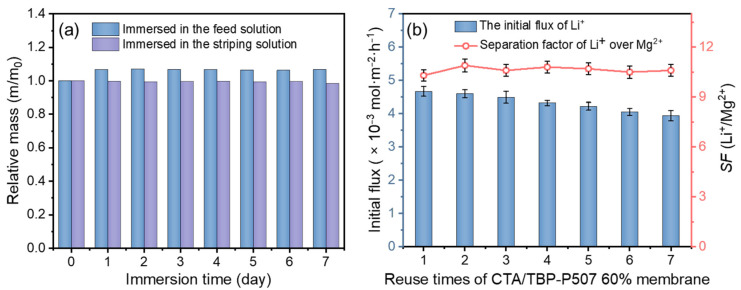
(**a**) Mass change of PIM after immersion in the feed solution and the striping solution (m_0_: the initial mass, 119.5 ± 0.3 mg; weighing interval: every 24 h); (**b**) reusability performance of the PIM system (feed solution: 0.1 mol/L LiCl, 4.0 mol/L MgCl_2_ and 0.13 mol/L FeCl_3_; test time: 24 h for one cycle; *n* = 5).

Stability and reusability are essential indicators for the industrial application of PIMs. The stability of the PIM was evaluated by the mass loss proposed in the literature [44]. Specifically, the fresh CTA/P507-TBP60% membrane (3 cm × 3 cm) was also immersed in 50 mL of feed solution or striping solution for 7 days, and the mass change was monitored every day to determine the leakage rate of the carrier. The corresponding results are shown in Figure 9a. It can be seen that the mass of the membrane immersed in the feed solution increased by about 7% on the first day. This is because the cations in the aqueous phase were extracted into the membrane phase, and the extraction process reached a plateau within 24 h owing to the limitation of carrier content. No obvious mass difference of the membrane was observed in the next 6 days. Similarly, when the membrane was immersed in the striping solution for 7 days, the mass change of the membrane was also negligible. These results indicate that the CTA-based PIMs we developed exhibit excellent stability. 

In addition, transport experiments were continuously carried out for seven cycles by reusing the same membrane (CTA/P507-TBP60%) to evaluate the reusability and service time of the membrane. The experimental conditions were described in Section 2.4. The transport time for one cycle was 24 h, and the solutions in both compartments were refreshed after each cycle. The initial flux of Li^+^ and the *SF*(Li^+^/Mg^2+^) of the membrane were calculated, and the results are presented in Figure 9b. The Li^+^ flux exhibits a slight decline over seven consecutive cycles; the values for the first cycle and the seventh cycle were 4.67 × 10^−3^ mol·m^−2^·h^−1^ and 3.95 × 10^−3^ mol·m^−2^·h^−1^, respectively. The *SF*(Li^+^/Mg^2+^) is almost unchanged over seven consecutive cycles, and the values were always above 10. These experimental results demonstrate that the PIM exhibits excellent stability. Therefore, the PIMs with CTA as the polymer matrix and P507-TBP as the Li^+^ transport carrier is suitable for effective lithium extraction from brines. 

#### 3.2.4. Ions Transport Kinetics across the PIM

**Figure 10:**
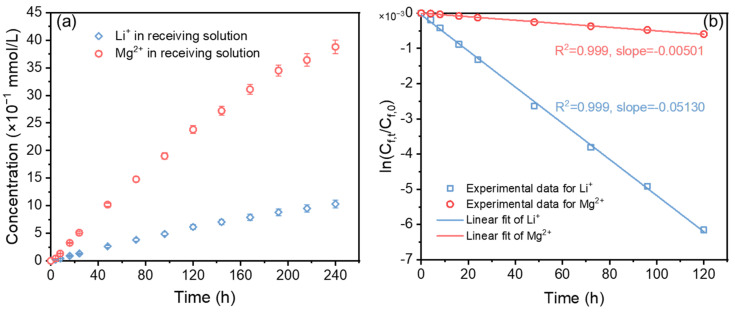
The results of the long-term transport experiment: (**a**) the concentrations of Li^+^ and Mg^2+^ in the receiving solution; (**b**) the kinetics plots of ln(*C_f,t_*/*C_f,0_*) vs. time; membrane: CTA/P507-TBP60%; feed solution: 0.1 mol/L of LiCl, 4.0 mol/L of MgCl_2_ and 0.13 mol/L of FeCl_3_; test time: 240 h; *n* = 5.

The kinetic properties of ion transport across the PIM play an important role in guiding the industrial application of the membrane. Here, to study the transport kinetics of Li^+^ and Mg^2+^ across CTA/P507-TBP60%, a transport experiment for 240 h was carried out, and the Li^+^ and Mg^2+^ in the receiving solution were detected periodically. It can be observed from Figure 10a that the Li^+^ and Mg^2+^ concentrations in the receiving solution increase steadily and still show an increasing trend at 240 h, demonstrating that Li^+^ was continuously transferred from the feed solution to the receiving solution during the experiment. The plot of ln(*C_f,t_*/*C_f,0_*) versus time for the transport of Li^+^ and Mg^2+^ across the PIM was shown in Figure 10b, and the resulting linear relationship indicates that the transport process follows Fick’s first law [45]. From the slope and Equation (1), the permeability coefficients of the Li^+^ and Mg^2+^ transport through CTA/P507-TBP60% are 4.76 × 10^−5^ m/h and 4.25 × 10^−6^ m/h, respectively. The value of Li^+^ is considerably larger than that of Mg^2+^, implying that the PIM we developed exhibits excellent selectivity for lithium. Determined by Equations (2) and (3), the initial flux of Li^+^ and the *SF*(Li^+^/Mg^2+^) are 4.76 × 10^−3^ mol·m^−2^·h^−1^ and 10.2, respectively. The Mg/Li molar ratio decreases from 40 in the feed solution to 3.9 in the receiving solution. 

In addition, the separation performance comparison of different liquid membranes is shown in Table 2. The CTA/P507-TBP60% membrane exhibits higher Li^+^ selectivity than most other membranes, and the permeability is comparable to other PIM membranes reported in the literature. However, it can be seen that the permeability of PIM is poor compared to the supported liquid membrane, which may be due to the restricted mobility of the extraction carrier in the PIM membrane and the different carriers used in these membranes. 

## 4. Conclusions

In this work, CTA-based PIMs containing P507 and TBP as extraction carriers were elaborated to recover Li^+^ from brines with a high Mg/Li mole ratio. The compatibility between CTA and P507-TBP is good, and the PIMs can be easily synthesized by the casting method. The PIMs can be used for lithium and magnesium separation in a broad range of Mg/Li mole ratios, and the Li^+^ is easily stripped from the PIM to the receiving solution using water. When the feed solution was synthetic brines, the CTA/P507-TBP60% membrane exhibited excellent Li^+^ selectivity (Li/Na = 13.2, Li/K = 32, Li/Mg = 21.8). The P507-TBP extraction system without involving concentrated hydrochloric acid eluents could help to keep the stability of PIMs. In the seven times’ reused transport experiments, the CTA/P507-TBP60% membrane retained excellent selectivity and permeability, showing a potential for long-term operation. The strategy of integrating P507-TBP extracting carriers into PIMs could help develop sustainable and highly stable PIMs for extracting lithium from natural salt-lake brines.

## Figures and Tables

**Table 1:** The compositions and thicknesses of PIMs.

Membranes	CTA	P507-TBP (*v*/*v*: 3/1)	Thickness (μm)
CTA/P507-TBP20%	0.2 g	0.05	72 ± 3
CTA/P507-TBP30%	0.2 g	0.086	81 ± 6
CTA/P507-TBP40%	0.2 g	0.133	97 ± 4
CTA/P507-TBP50%	0.2 g	0.2	112 ± 3
CTA/P507-TBP60%	0.2 g	0.3	132 ± 4
CTA/P507-TBP70%	0.2 g	0.467	165 ± 3

**Table 2:** Comparison of separation performance of different liquid membranes for Li^+^ extraction.

Membranes	Feed Solution	*J_0_*, (mol·m^−2^·h^−1^)	Recovery Percentage (%)	Separation Factor	Refs
PIM: PVC/c-TBP-50%	0.2 mol/L LiCl + 3.0 mol/L MgCl_2_	Li^+^: 8.12 × 10^−4^	25	Li/Mg: 176	[33]
PIM: CTA/TBP-[C_4_mim] [NTf_2_] 40%	7.2 mmol/L LiCl + 2.06 mmol/L MgCl_2_	Li^+^: 3.2 × 10^−3^	19	Li/Mg: 2.2	[34]
PIM: CTA + TTA + TOPO	2.9 mmol/L LiCl + 0.87 mmol/L NaCl + 0.51 mmol/L KCl	Li^+^: 5.79 × 10^−3^	88	Li/Na: 54.3	[31]
				Li/K: 50.6	
SLM: PVDF/TBP-[C_4_mim] [NTf_2_]	12.83 mmol/L LiCl + 5.17 mmol/L MgCl_2_	Li^+^: 50.7 × 10^−3^	46	Li/Mg: ~1.6	[17]
SLM: TBP + FeCl_3_ system	0.15 mol/L LiCl + 2.06 mol/L MgCl_2_ + 0.20 mol/L FeCl_3_	--	48	Li/Mg: ~14.5	[46]
SLM: TBP + ClO_4_^-^system	0.15 mol/L LiClO_4_ + 2.06 mol/L MgCl_2_	--	41	Li/Mg: ~8.2	[46]
PIM: CTA/P507-TBP60%	0.1 mol/L LiCl + 4.0 mol/L MgCl_2_ + 0.13 mol/L FeCl_3_	Li^+^: 4.76 × 10^−3^	55	Li/Mg: 10.2	This work
PIM: CTA/P507-TBP60%	0.1 mol/L LiCl + 2.0 mol/L NaCl + 0.5 mol/L KCl + 1.0 mol/L MgCl_2_ + 0.13 mol/L FeCl_3_	Li^+^: 3.86 × 10^−3^	47	Li/Na: 13.2	This work
				Li/K: 32.0	
				Li/Mg: 21.8	
